# Patient preference for intraoperative opioid strategy and early postoperative recovery after noncardiac surgery: a prospective partially randomised patient-preference trial (the PERFECT trial)

**DOI:** 10.1016/j.bjao.2026.100867

**Published:** 2026-10-26

**Authors:** Yann Gricourt, Nancy M. Boulos, Amelie Delaporte, Maziar M. Nourian, Daniel Pearce, Ido Neuman, Reza Borna, Josue Chirinos, Kenneth Lin, Justin Mitchell, Sebastian Kahf, Theodora Wingert, Tristan Grogan, David Boldt, Maxime Cannesson, Patrice Forget, Alexandre Joosten

**Affiliations:** 1 Department of Anaesthesiology and Perioperative Medicine, David Geffen School of Medicine, University of California, Los Angeles, Los Angeles, CA, USA; 2 Department of Anaesthesiology and Perioperative Medicine, Nîmes University Hospital, Nîmes, France; 3 IMAGINE UR UM 103, Montpellier University, Anaesthesia Critical Care, Emergency and Pain Medicine Division, Nîmes University Hospital, Nîmes, France; 4 Department of Medicine Statistics Core, David Geffen School of Medicine, University of California Los Angeles, Los Angeles, CA, USA; 5 Aberdeen Centre for Arthritis and Musculoskeletal Health (Epidemiology Group), Institute of Applied Health Sciences, School of Medicine, Medical Sciences and Nutrition, Aberdeen, UK; 6 Anaesthesia Department, NHS Grampian, Aberdeen, UK; 7 Pain and Opioids After Surgery (PANDOS) European Society of Anaesthesiology and Intensive Care (ID ESAIC_RG_PAND) Research Group, Brussels, Belgium

**Keywords:** opioid-free anaesthesia, opioids, patient-centred care, patient preference, quality of recovery, shared decision making

## Abstract

**Background:**

Patient-centred care increasingly encourages patients to participate in decisions regarding their anaesthetic management. However, whether expressing a preference regarding their anaesthetic management is associated with improved postoperative recovery remains unknown. We evaluated the association between preference expression and quality of recovery after noncardiac surgery.

**Methods:**

In this prospective partially randomised patient-preference study conducted at two academic hospitals, adults undergoing minimally invasive abdominal, urologic, or gynaecologic surgery received standardised information on opioid-based and opioid-free anaesthesia, co-developed with patient partners. Participants expressing a preference received their preferred strategy, whereas those without a preference were randomised 1:1 to either strategy. The primary outcome was the Quality of Recovery-15 (QoR-15) score on postoperative day (POD) 1.

**Results:**

Among 220 analysed participants, 177 (80.5%) expressed a preference, and 43 (19.5%) underwent random allocation. Mean [standard deviation] QoR-15 scores on POD 1 were higher among participants expressing a preference than among randomised participants (108.8 [18.2] *vs* 100.8 [23.5]), with an unadjusted mean difference of 7.9 points (95% confidence interval [CI] 1.5–14.4; *P*=0.017). The adjusted marginal mean difference was 7.1 points (95% CI 0.3–13.9; *P*=0.042). Scores on the QoR-15 also remained higher on POD 2. Sensitivity analyses showed consistent effect estimates but attenuated statistical significance. Secondary outcomes, including postoperative nausea and vomiting and bradycardia, were similar between groups.

**Conclusions:**

Expression of a preference regarding intraoperative opioid management was associated with better reported early postoperative patient-reported recovery. Given the non-randomised comparison and limited precision, -generating and support further investigation into the role of patient-preference expression in perioperative recovery.

**Trial registration:**

The full, long-term study is pre-registered at ClinicalTrials.gov (identifier, NCT06855641; first posted, 3 March 2025; Alexandre Joosten). https://clinicaltrials.gov/study/NCT06855641.

**Editor’s key points**

• Although shared decision making is increasingly embedded in perioperative care frameworks, whether patient preference expression itself, independent of the strategy received, influences postoperative recovery has not previously been examined in a prospective study.
• The partially randomised patient-preference design allows simultaneous analysis of a preference cohort and a randomised cohort, a methodological approach not previously applied in perioperative medicine.
• The observed mean Quality of Recovery-15 difference of 7.9 points (95% confidence interval, 1.5–14.4) on the first postoperative day indicates a potentially clinically relevant difference.
• Secondary outcomes including postoperative nausea and vomiting and bradycardia did not differ between opioid-based and opioid-free anaesthesia groups, suggesting that the treatment strategy chosen was not the principal driver of the recovery difference.
• The non-randomised nature of the preference comparison limits causal inference; the authors acknowledge this explicitly, and a pre-registered long-term trial is underway.

Involving patients in shared decision making is widely recognised as a cornerstone of patient-centred perioperative care.^1^, ^2^, ^3^ Across a range of medical specialities, active patient participation in treatment decisions has been associated with reduced decisional conflict, greater satisfaction, improved treatment adherence, and, in some settings, better patient-reported outcomes.^4^, ^5^, ^6^, ^7^ Despite this, anaesthesiology remains largely clinician driven, with limited patient involvement in selecting anaesthetic strategies.^8^^,^^9^ Whether expressing a clear preference for general anaesthesia after receiving balanced information is associated with postoperative recovery remains largely unknown.

The importance of patient participation may extend beyond ethical considerations alone. Evidence from patient-preference trials suggests that treatment preferences, expectations, perceived autonomy, and active engagement in care can influence clinical outcomes independently of the treatment received.^10^, ^11^, ^12^ Indeed, studies in musculoskeletal disorders, rehabilitation, and mental health suggest that accommodating patients’ treatment preferences may enhance treatment satisfaction, therapeutic alliance, and patient engagement, while reducing attrition and potentially improving patient-reported outcomes compared with those assigned to a non-preferred strategy.^10^^,^^13^, ^14^, ^15^ However, in perioperative medicine, evidence remains scarce. In a randomised trial of women undergoing Caesarean delivery, offering a choice of postoperative analgesic regimen did not reduce opioid consumption or pain scores but was associated with greater patient satisfaction.^16^ These findings suggest that patient choice may influence patient-reported outcomes more than traditional analgesic endpoints.

One relevant perioperative decision is intraoperative opioid use. Opioid-based anaesthesia (OBA) remains widely used,^17^ but concerns regarding opioid-related adverse effects, including postoperative nausea and vomiting (PONV), respiratory depression, ileus, urinary retention, and delayed recovery, have stimulated interest in opioid-free anaesthesia (OFA) approaches.^18^^,^^19^ OFA typically combines regional anaesthesia techniques and non-opioid agents such as ketamine, dexmedetomidine, and lidocaine to provide adequate antinociception while avoiding intraoperative opioid administration.^20^^,^^21^

Although randomised trials and meta-analyses have compared OFA and OBA, results remain inconsistent and debated.^22^, ^23^, ^24^, ^25^ Although studies have confirmed reductions in PONV,^26^^,^^27^ improvements in broader patient-centred recovery outcomes have generally been modest.^21^^,^^28^, ^29^, ^30^ This raises the possibility that factors beyond the pharmacological regimen itself may contribute to postoperative recovery experiences.

Surveys suggest that many surgical patients wish to participate in anaesthetic decision making and value being informed about available anaesthetic options.^31^ However, to the best of our knowledge, no prospective study has evaluated whether expressing a preference regarding anaesthetic management is associated with postoperative recovery.

The Patient Preference for Intraoperative Opioid Strategy and Postoperative Effect (PERFECT) study was designed to evaluate whether expression of a treatment preference regarding intraoperative opioid strategy was associated with early postoperative quality of recovery (QoR) after elective minimally invasive noncardiac surgery. Because all participants received the same standardised decision aid and dedicated discussion with the study investigators, the exposure of interest was preference expression rather than shared decision making itself.

We hypothesised that participants who expressed a preference for their anaesthetic strategy would report better postoperative recovery than those who did not express a preference and were subsequently randomised, regardless of the anaesthetic regimen ultimately received.

## Methods

### Study design and ethics

Ethics approval was obtained from the University of California, Los Angeles (UCLA) (IRB-24-5789, 24 February 2025), and the trial was registered at ClinicalTrials.gov (NCT06855641; first posted 3 March 2025) before enrolment. The protocol has been previously published.^32^ Although the protocol title referred to a ‘randomised factorial design trial’, the study design implemented throughout was a partially randomised patient-preference trial, as described in the protocol methods. The use of the term ‘factorial’ in the protocol title reflected imprecise terminology rather than a change in study design. In brief, the PERFECT study was a prospective partially randomised patient-preference trial conducted at two academic medical centres within the UCLA health system (Ronald Reagan UCLA Medical Centre and UCLA Santa Monica Medical Centre). Given the hybrid design of this study, reporting was informed using both the Strengthening the Reporting of Observational Studies in Epidemiology statement for the observational comparison^33^ and the Consolidated Standards of Reporting Trials statement for the randomised component of the trial.^34^ Importantly, all participants provided a written informed consent before anaesthesia.

### Participants

Adults aged 18 yr or older undergoing elective minimally invasive abdominal, urologic, or gynaecologic surgery (robotic or laparoscopic approach) were eligible. Exclusion criteria included chronic opioid use, chronic pain requiring ongoing analgesic treatment, pregnancy or breastfeeding, severe psychiatric disease, planned epidural analgesia, contraindication to study medications, inability to provide an informed consent, or inability to complete study questionnaires in English.

### Preference assessment and group allocation

One week before surgery, participants received a dedicated patient decision aid developed collaboratively by anaesthesiologists and patient partners describing potential advantages and disadvantages of OBA and OFA^35^ (Supplementary Material 1). On the day of surgery, investigators met each eligible patient to provide additional explanations about both strategies before obtaining a written informed consent. Investigators also answered participants’ questions but did not encourage or discourage either strategy. To support preference expression, no standardised script was used, and discussions were pragmatically tailored to participants’ individual questions. This final discussion took place in the preoperative area, after which participants were invited to indicate their preference. Participants expressing a preference received their preferred strategy, whereas those reporting no preference or remaining uncertain were randomised.

This design was chosen to evaluate the association between treatment preference and postoperative recovery while preserving randomisation among participants without a treatment preference. Participants were also classified into four groups: OBA with expressed preference (OBA-P), OFA with expressed preference (OFA-P), randomised OBA (OBA-R), and randomised OFA (OFA-R). Because all participants received the same decision aid and standardised discussion, the comparison of interest was between participants who expressed a treatment preference and those who did not, rather than between shared decision making and usual care. The preference process was assessed on postoperative day (POD) 1 using a piloted questionnaire, including two prespecified rating scale items evaluating decision process feeling and difficulty in choosing between OFA and OBA.

Anaesthesia clinicians, study investigators, and participants who expressed a preference were aware of treatment allocation. Participants undergoing random allocation, surgeons, postoperative healthcare providers, outcome assessors, and the study statistician remained blinded to anaesthetic assignment throughout data collection and primary analyses.

#### Anaesthetic strategies

In both groups, anaesthesia maintenance and haemodynamic management (fluid and vasopressor) were left to the anaesthesiologist’s discretion. Wound infiltration by surgeons was the standard of care in our centres, whereas fascial plane blocks with bupivacaine were provided preoperatively by a dedicated regional anaesthesia team, upon request by the anaesthesiologist, for pain management. Unless contraindicated or considered clinically inappropriate by the treating anaesthesiologist, multimodal analgesia included i.v. acetaminophen and an NSAID. Omission of acetaminophen or an NSAID for clinical reasons was permitted and was not considered a protocol deviation.

Acetaminophen 1 g i.v. was administered approximately 30 min before induction (pre-emptive analgesia). An NSAID (ketorolac 15–30 mg i.v. or ibuprofen 400–600 mg orally) was given after surgical closure as preventive analgesia, unless contraindicated. Systematic PONV prophylaxis consisted of i.v. dexamethasone 10 mg at induction and ondansetron 4 mg before emergence. Additional medications, such as droperidol or a scopolamine patch, were administered according to the Apfel score. In the postanaesthesia care unit (PACU), pain was managed by registered nurses under the local protocol using i.v. opioids (tramadol or titrated hydromorphone) or oral oxycodone when tolerated, until achieving a numeric rating scale (NRS) score of less than 3/10.

Participants in the OFA strategy received no intraoperative opioids and at least two of the following infusions to comply with the protocol: i.v. ketamine (0.5 mg kg^−1^ loading dose followed by a 0.2 mg kg^−1^ h^−1^ continuous infusion), i.v. lidocaine (100 mg loading dose followed by a 1.5 mg kg^−1^ h^−1^ continuous infusion), i.v. dexmedetomidine (0.5 μg kg^−1^ over 10 min followed by a continuous infusion at 50% of the loading dose per hour), or i.v. magnesium sulphate (40 mg kg^−1^ loading dose). In the OBA strategy, participants received lidocaine (loading dose 1 mg kg^−1^) in combination with opioids at the anaesthesiologist’s discretion (fentanyl 100 μg at induction with additional 50 μg boluses or, alternatively, remifentanil or hydromorphone).

Protocol adherence was assessed according to delivery of the assigned anaesthetic strategy. OBA was defined as an anaesthetic plan without routine intraoperative opioid administration and using at least two of the protocol-specified non-opioid agents described earlier. When clinically necessary because of haemodynamic instability or uncontrolled nociception, rescue opioid administration was permitted and was recorded separately rather than considered automatic crossover. OBA was defined as planned intraoperative opioid administration without non-opioid adjuncts, except for lidocaine at induction to reduce propofol injection pain; standard multimodal analgesic components permitted in both strategies were not considered protocol deviations. Participants with deviations after treatment initiation were included in the primary analysis but excluded from the per-protocol analysis. No nociception monitoring device was used during the study.

### Main outcomes and measures

The primary outcome was the Quality of Recovery-15 (QoR-15) total score on POD 1,^36^ comparing participants who expressed a preference with those who did not. This score includes 15 questions covering five key dimensions of postoperative recovery: physical comfort, emotional state, psychological support, physical independence, and pain management. Each item is scored from 0 to 10, with the sum ranging from 0 to 150. Higher scores reflect better recovery. The questionnaire was completed by participants themselves in the hospital or by blinded research assessors via phone call if the participant had already been discharged home.

Secondary outcomes included QoR on POD 2, anaesthesia satisfaction assessed using the Bauer questionnaire on POD 1,^37^ health-related quality of life using the EuroQol 5-Dimension 5-Level questionnaire (EQ-5D-5L) score on POD 30,^38^ intraoperative bradycardia incidence, and PONV. Exploratory outcomes were safety outcomes such as adverse effects related to the intraoperative anaesthesia protocol, hospital length of stay, postoperative pain intensity at rest assessed using an NRS within 48 h, incidence of postoperative surgical complications (Clavien–Dindo classification), hospital readmission, and days at home alive within 30 days (DAH30),^39^ all assessed on POD 30.

### Data collection and monitoring

All data were collected from UCLA electronic medical records (Epic Systems, Verona, WI, USA) or postoperative patient phone calls. Baseline demographic variables, medical history, and factors potentially influencing anaesthesia preference (history of opioid use, substance abuse or chronic pain, previous PONV, previous abdominal surgery, and type of scheduled surgery) were recorded. Patient feedback on the decision-making process, including preoperative anxiety level, was also collected. No data and safety monitoring board was required by the ethics committee for this study.

### Sample size and statistical analysis

At the time of protocol development, no previous studies had evaluated the association between preference expression and postoperative recovery. Therefore, sample size calculations were informed by contemporary studies comparing OFA and OBA using the QoR-15 score. Based on published data,^29^ demonstrating a between-group difference of approximately 6 points in QoR-15 scores and considering the established minimal clinically important difference (MCID) of 8 points,^40^ a sample size of 90 participants per primary comparison group was estimated to provide approximately 90% power to detect a clinically meaningful difference using a two-sided α level of 0.05. Allowing for loss to follow-up and protocol deviations, the planned sample size was 240 participants. Because preference expression was not randomised and group sizes were expected to be unequal, the study was primarily designed to estimate effect sizes and assess feasibility rather than establish definitive causal associations.

Patient characteristics and outcomes were summarised by group using means (standard deviations [sds]), medians (interquartile ranges), or frequencies (%), as appropriate.

The primary analysis evaluated the association between preference expression (yes *vs* no) and POD 1 QoR-15 using multivariable linear regression. The prespecified model included preference expression, anaesthetic strategy (OFA *vs* OBA), their interaction, age, sex, history of opioid use, chronic pain, substance use disorder, PONV history, previous abdominal surgery, surgical duration, and five surgical-category indicator terms (gastrointestinal, colorectal, urologic, gynaecologic, and hernia repair). Covariates were selected *a priori* based on clinical relevance and anticipated relationships with preference expression and postoperative recovery, rather than on data-driven variable selection. The model estimated 17 parameters, including the intercept.

Because the model included a preference-expression × anaesthetic-strategy interaction, the primary estimand was the adjusted marginal mean difference in QoR-15 between preference-expression and no-preference-expression groups, averaged equally across OBA and OFA. Accordingly, the regression coefficient for preference expression alone represents the conditional contrast within the reference anaesthetic strategy and is not the overall primary estimand. The adjusted primary model used complete cases for the outcome and all model covariates (*N*=213). An exploratory marginal comparison of OFA with OBA, averaged across preference-expression strata, was also estimated from the primary model. Because the anaesthetic strategy was not randomised among participants who expressed a preference, this comparison was considered descriptive/exploratory rather than a causal treatment-effect estimate.

The primary analysis used a modified intention-to-treat (ITT) analytic population. Of the 240 enrolled participants, 16 withdrew before postoperative assessment, and four participants did not receive the assigned study intervention before anaesthesia (three OBA-P and one OFA-R); therefore, these participants did not contribute to the primary POD 1 analysis. The resulting primary analytic population comprised 220 participants. Participants with protocol deviations after initiation of the assigned anaesthetic strategy were included in the primary analysis but excluded from the prespecified per-protocol analysis.

To explore factors associated with preference expression, univariable and multivariable logistic regression analyses were performed using preference expression as the dependent variable. These exploratory models were intended to describe measured characteristics associated with preference expression and were not used to establish comparability or exchangeability between the non-randomised groups. Sensitivity analyses evaluated the robustness of the primary findings using alternative weighting approaches and variance estimators. To assess model robustness and potential overfitting, *post hoc* parsimonious sensitivity models were fitted using the same complete-case sample. Model diagnostics included assessments of influential observations using Cook’s distance and studentised residuals, along with heteroskedasticity, collinearity, and non-linearity of the continuous covariates. Secondary outcomes were analysed descriptively and using unadjusted comparisons because the study was not powered for these analyses. A prespecified per-protocol analysis was also performed among participants with complete adherence to the allocated anaesthetic strategy. Missing outcome data were not imputed. Analyses of each endpoint were based on participants with available outcome data for that endpoint; the adjusted primary model additionally required complete data for all model covariates. All statistical tests were two sided, and *P*<0.05 was considered statistically significant. Complete regression model specifications and parameter estimates for the primary and secondary outcomes are presented in Supplementary Table 1.

## Results

### Participant characteristics

Between 24 March and 16 June 2025, 240 of 397 eligible participants were enrolled (Fig. 1). Of these, 194 (80.8%) expressed a preference for intraoperative opioid management, whereas 46 (19.2%) did not express a preference and were randomly allocated. Sixteen participants subsequently withdrew before postoperative assessment, and four did not receive the assigned study intervention before anaesthesia, leaving 220 participants in the primary analytic population: 177 in the preference-expression group and 43 in the no-preference-expression group. QoR-15 data on POD 2 were available for 203 participants, including 165 of 177 (93.2%) in the preference-expression group and 38 of 43 (88.4%) in the no-preference-expression group (*P*=0.337). EQ-5D-5L assessments at one month were available for 165 participants, including 134 of 177 (75.7%) and 31 of 43 (72.1%), respectively (*P*=0.695). Therefore, follow-up availability did not differ significantly by preference-expression status. Measured baseline demographic, clinical, and surgical characteristics were also generally similar between participants with and without available follow-up data. Missing follow-up outcomes were not imputed. Among analysed participants, 139 (63.2%) were women, and 196 (89.1%) had previously undergone general anaesthesia. Baseline characteristics according to preference expression are presented in Table 1 and Supplementary Table 2.

**Fig 1:**
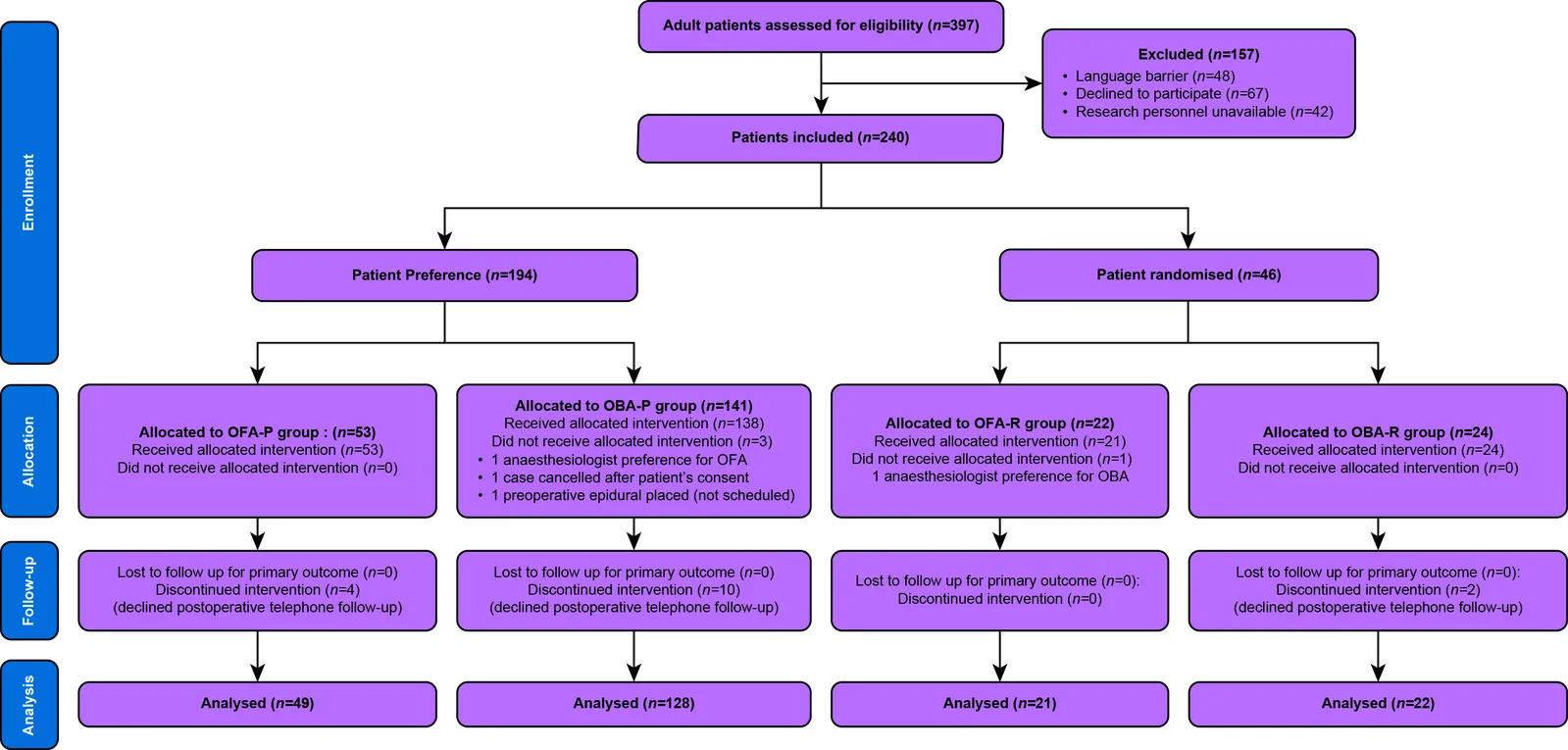
Consolidated Standards of Reporting Trials flow diagram. OBA, opioid-based anaesthesia; OFA, opioid-free anaesthesia; P, preference; R, randomised.

**Table 1:** Baseline characteristics. Data are presented as mean [standard deviation] or *n* (%). COPD, chronic obstructive pulmonary disease; PONV, postoperative nausea and vomiting. ∗Defined as any level of worry about receiving general anaesthesia (any response other than ‘not at all worried’).

Variables	Preferenceexpression(*n*=177)	No preferenceexpression(*n*=43)
Age, yr	56 [15]	51 [15]
Female sex	108 (61)	31 (72)
Weight, kg	81 [18]	87 [25]
BMI, kg m^−2^	28 [6]	30 [8]
College education or greater	135 (79)	34 (83)
Ethnicity		
White	118 (67)	22 (51)
Hispanic	19 (11)	9 (21)
Asian	21 (12)	4 (9)
African American	6 (3)	2 (5)
Others	11 (6)	6 (14)
Type of surgery		
Gastrointestinal	21 (12)	12 (28)
Colorectal	10 (6)	5 (12)
Hernia repair	27 (15)	6 (14)
Urologic	55 (31)	6 (14)
Gynaecologic	65 (37)	16 (37)
ASA physical status score		
I	12 (7)	3 (7)
II	89 (50)	20 (47)
III	75 (42)	20 (47)
IV	1 (0.6)	0 (0)
Medical history		
High blood pressure	74 (42)	16 (37)
Dyslipidaemia	83 (47)	17 (40)
Coronary artery disease	23 (13)	3 (7)
Diabetes mellitus	25 (14)	9 (21)
COPD/asthma	21 (12)	5 (12)
Sleep apnoea	25 (14)	7 (16)
Chronic renal failure	7 (4)	1 (2)
Active cancer	73 (41)	7 (16)
Substance use disorder	50 (28)	10 (23)
Previous chronic pain	16 (9)	5 (12)
Previous chronic opioid use	23 (13)	4 (9)
Previous general anaesthesia	157 (89)	38 (88)
Previous abdominal surgery	106 (60)	23 (54)
Previous PONV	29 (16)	5 (12)
Current medications		
Antidepressant	28 (16)	9 (21)
Anxiolytic	14 (8)	6 (14)
Non-opioid analgesics	67 (38)	11 (26)
Preoperative anxiety related to		
General anaesthesia∗	100 (58)	23 (54)
Opioid exposure	101 (59)	25 (58)

#### Preference expression and decision making

Doubts or hesitation during the decision process were reported by only 11 participants (6.4%) in the preference group and 1 (2.4%) in the randomised group (*P*=0.557). Reasons for preferring OFA or OBA or expressing no preference are also presented in Supplementary Fig. 1. Among the 177 participants expressing a preference, 128 (72.3%) chose OBA and 49 (27.7%) chose OFA. This decision was reported as not at all difficult by 20 OFA (43.5%) and 46 OBA participants (38.0%) (overall *P*<0.001). Importantly, educational attainment (college education or greater), previous opioid exposure, previous opioid experiences, opioid-related knowledge, knowledge of general anaesthesia, confidence during the decision-making process, and trust in the anaesthesia team were similar between participants who expressed a preference and those who underwent randomisation (all *P*>0.05). Exposure to the opioid crisis through relatives or acquaintances was also similar between groups (19.0% *vs* 19.2%; *P*=0.984). General anaesthesia-related anxiety was reported by 100 participants (58%) in the preference-expression group and 23 (54%) in the randomised group (Table 1).

### Intraoperative characteristics

Intraoperative data, including surgical details, anaesthetic medications, and postoperative pain management, are presented in Table 2. No major intraoperative protocol-related adverse events were reported.

**Table 2:** Intraoperative data according to the allocated groups. Data are presented as mean [standard deviation] or *n* (%). OBA, opioid-based anaesthesia; OBA-P, opioid-based anaesthesia with expressed preference; OBA-R, randomised opioid-based anaesthesia; OFA, opioid-free anaesthesia; OFA-P, opioid-free anaesthesia with expressed preference; OFA-R, randomised opioid-free anaesthesia; PONV, postoperative nausea and vomiting.

Characteristics	Preference expression		No preference expression	
	OFA-P (*n*=49)	OBA-P (*n*=128)	OFA-R (*n*=21)	OBA-R (*n*=22)
Length of surgery, min	163 (91)	156 (76)	126 (83)	149 (75)
Surgical approaches				
Laparoscopic assisted	14 (29)	40 (31)	12 (57)	10 (46)
Robotic assisted	35 (71)	88 (69)	9 (43)	11 (50)
Conversion to open surgery	0	0	0	2 (9)
Estimated blood loss, ml	121 (182)	97 (204)	49 (75)	67 (60)
Length of anaesthesia, min	238 (104)	227 (86)	193 (95)	214 (79)
Anaesthesia maintenance				
Sevoflurane	43 (88)	117 (92)	19 (90)	18 (82)
Total i.v. propofol	2 (4)	3 (2)	1 (5)	0 (0)
Combination of both	4 (8)	8 (6)	1 (5)	4 (18)
Depth of anaesthesia monitoring	28 (57)	46 (36)	8 (38)	6 (27)
Midazolam	30 (61)	90 (70)	15 (71)	17 (77)
Antibiotics	49 (100)	122 (95)	19 (91)	21 (96)
Anaesthetic medications				
Ketamine	45 (92)	29 (23)	19 (91)	7 (32)
Dose in mg	65 (34)	13 (26)	58 (37)	14 (25)
Lidocaine bolus	47 (96)	118 (92)	20 (95)	21 (96)
Dose in mg	83 (33)	65 (30)	73 (29)	71 (29)
Lidocaine continuous infusion	19 (39)	3 (2)	6 (29)	1 (5)
Dose in mg	90 (135)	7 (46)	93 (192)	6 (28)
Magnesium	18 (37)	1 (0.8)	4 (19)	0
Dose in mg	939 (1313)	9 (97)	476 (1031)	0
Dexmedetomidine	44 (90)	25 (20)	17 (81)	5 (23)
Dose in μg	62 (57)	6 (15)	50 (50)	6 (11)
Fentanyl	1 (2)	118 (92)	1 (5)	20 (91)
Dose in μg	2 (14)	119 (66)	5 (22)	121 (71)
Remifentanil	0	2 (2)	0	0
Dose in μg	0	19 (162)	0	0
Hydromorphone	5 (10)	91 (71)	0	15 (68)
Dose in mg	0.1 (0.2)	0.5 (0.5)	0	0.7 (0.7)
Dexamethasone	47 (96)	124 (97)	20 (95)	22 (100)
Dose in mg	8.2 (2.7)	8 (3)	8 (4)	8 (3)
Protocol compliance	44 (90)	80 (63)	20 (95)	12 (55)
Unplanned protocol interruption	1 (2)	2 (2)	1 (5)	0 (0)
Multimodal analgesia				
Fascial plane block	7 (14)	19 (15)	6 (29)	6 (27)
Wound infiltration	42 (86)	109 (85)	16 (76)	18 (82)
Acetaminophen	45 (92)	115 (90)	19 (91)	21 (96)
NSAID	25 (51)	49 (38)	12 (57)	11 (50)
PONV prophylaxis	49 (100)	127 (100)	20 (100)	22 (100)
Number of anti-emetic drugs received				
≤2	28 (57)	86 (67)	11 (52)	10 (46)
>2	21 (43)	42 (33)	10 (48)	12 (55)
Postoperative destination				
Surgical ward	3 (6)	22 (17)	3 (14)	5 (23)
Transitional recovery unit	25 (51)	63 (49)	10 (48)	8 (36)
Home discharge	21 (43)	43 (34)	8 (38)	9 (41)

### Primary outcome

On POD 1, participants expressing a preference reported higher QoR-15 scores than participants who underwent random allocation (mean [sd] 108.8 [18.2] *vs* 100.8 [23.5]), corresponding to an unadjusted mean difference of 7.9 points (95% confidence interval [CI] 1.5–14.4; *P*=0.017) (Table 3 and Fig. 2). The adjusted primary regression included 213 participants with complete POD 1 outcome and covariate data. After adjustment for anaesthetic strategy and prespecified covariates, preference expression remained associated with higher QoR-15 scores (adjusted marginal mean difference 7.1 points; 95% CI 0.3–13.9; *P*=0.042). Because the model included a preference-expression × anaesthetic-strategy interaction, the 7.1-point estimate represents the marginal preference *vs* no-preference contrast, averaged across anaesthetic strategies. There was no evidence that this association differed by anaesthetic strategy (interaction estimate 4.86 points; 95% CI −8.37 to 18.09; *P*=0.472). The adjusted preference *vs* no-preference contrast was 4.67 points within OBA (95% CI −4.18 to 13.53; *P*=0.299) and 9.53 points within OFA (95% CI −0.64 to 19.71; *P*=0.066). In an exploratory marginal comparison across preference-expression strata, POD 1 QoR-15 did not differ between OFA and OBA (adjusted mean difference −1.27 points; 95% CI −7.96 to 5.42; *P*=0.708). Results were similar using HC3 robust standard errors (95% CI −9.33 to 6.79; *P*=0.757). In sensitivity analyses using alternative variance estimators and weighting approaches, effect estimates remained directionally consistent, although statistical evidence was attenuated (*P*-values ranging from 0.08 to 0.13). Confidence intervals remained wide, reflecting limited precision owing to group imbalance. In the prespecified per-protocol analysis, the adjusted mean difference was 9.1 points (95% CI 1.0–17.2; *P*=0.028). Because the per-protocol population excludes participants according to post-allocation adherence, this analysis was considered supportive and is more susceptible to selection bias than the primary analysis. Postoperative QoR-15 score distributions across the four groups are presented in Supplementary Fig. 2. Observed mean [sd] POD 1 QoR-15 scores across the four study groups were 110.1 [18.4] for OFA-P, 108.2 [18.2] for OBA-P, 98.8 [25.0] for OFA-R, and 102.7 [22.4] for OBA-R. Parsimonious sensitivity models produced marginal preference-effect estimates of similar magnitude, ranging from 6.46 to 7.73 points. In a *post hoc* sensitivity analysis excluding the two boxplot-defined outliers in the preference-expression group, the adjusted marginal difference was 7.25 points (95% CI 0.67–13.83; *P*=0.031); the corresponding heteroskedasticity-consistent type 3 (HC3) robust standard errors was 7.25 points (95% CI −0.69 to 15.19; *P*=0.073).

**Table 3:** Primary outcome. Adjusted analysis used 213 complete cases, and identify or cross-reference the adjustment model. The values are means [standard deviation], n (%), or mean differences (95% CIs). POD, postoperative day; QoR-15, Quality of Recovery-15.

Outcomes	Preferenceexpression(*n*=177)	No preferenceexpression(*n*=43)	Mean difference(unadjusted effect)	*P*-value(unadjusted)	Mean difference(adjusted effect)	*P*-value(adjusted)
QoR-15 on POD 1	108.8 [18.2]	100.8 [23.5]	7.9 (1.5–14.4)	0.017	7.1 (0.3–13.9)	0.042
Subdimensions						
1. Physical comfort	35.3 [8.4]	32.7 [11.1]	2.6 (−0.4 to 5.6)	0.095	2.1 (−1.1 to 5.2)	0.193
2. Pain management	13.3 [3.9]	11.9 [4.3]	1.4 (0.04–2.7)	0.043	1.0 (−0.3 to 2.4)	0.124
3. Physical independence	11.5 [4.6]	9.8 [4.9]	1.6 (0.07–3.2)	0.040	2.2 (0.5–3.8)	0.012
4. Psychological support	18.7 [2.1]	18.0 [2.6]	0.6 (−0.1 to 1.4)	0.105	0.6 (−0.2 to 1.4)	0.166
5. Emotional state	30.2 [6.4]	28.3 [8.0]	1.9 (−0.3 to 4.2)	0.096	1.3 (−1.0 to 3.7)	0.274

**Fig 2:**
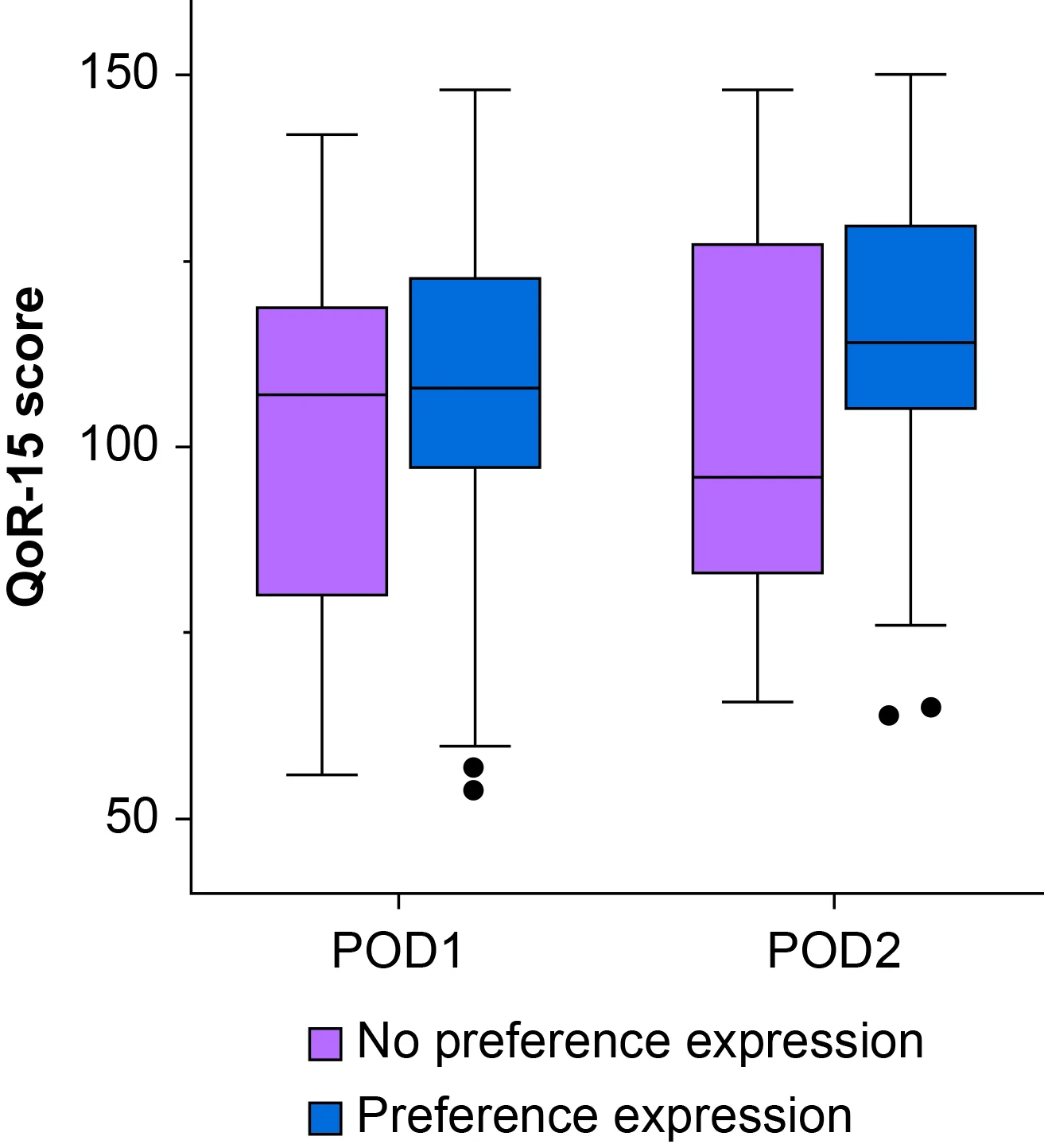
Distribution of postoperative QoR-15 scores on PODs 1 and 2 for preference expression *vs* no preference expression. Boxplots represent the interquartile range (25th to 75th percentile); horizontal lines indicate medians; whiskers extend to values within 1.5× the interquartile range. Dots beyond the whiskers represent boxplot-defined outliers; all observations were retained in the primary analysis. POD, postoperative day; QoR-15, Quality of Recovery-15. POD 1, 177 and 43; POD 2, 165 and 38, with the group order explicitly identified.

Subdomain-adjusted analyses indicated that higher QoR-15 scores among participants who expressed a preference were primarily driven by differences in the physical independence domain, whereas other recovery dimensions showed smaller, non-significant associations (Table 3). In the exploratory multivariable model, no measured baseline characteristic was independently associated with preference expression (Supplementary Table 3); however, this analysis was descriptive and does not establish comparability between the non-randomised groups. Finally, sensitivity analyses are presented in Supplementary Table 4. Full multivariable regression model results, including prespecified and sensitivity analyses using alternative weighting schemes and variance estimators, are presented in Supplementary Table 5.

### Secondary outcomes

On POD 2, among 203 participants with available QoR-15 data (165 preference expression and 38 no preference expression), mean [sd] scores were 115.6 [16.2] and 103.1 [23.4], respectively (*P*<0.001). Rates of PONV, intraoperative bradycardia, hypoxaemia, and postoperative opioid administration were similar between groups (Table 4). In the PACU, opioid consumption and requirements (79.1% *vs* 77.4%; *P*=0.810) were similar between groups. In detail, opioid consumption was lower in the OFA than in the OBA groups (Supplementary Table 6).

**Table 4:** Secondary outcomes. The values are means [standard deviation] and *n* (%). CI, confidence interval; EQ-5D-5L, EuroQol 5-Dimension 5-Level questionnaire; POD, postoperative day; PONV, postoperative nausea and vomiting; QoR-15, Quality of Recovery-15; VAS, visual analogue scale. PACU, Postanaesthesia Care Unit. ∗Bauer questionnaire: only ‘very satisfied’ responses are presented. ^†^Overall satisfaction is reported as a 0–10 numeric rating scale. ^‡^Among patients followed on POD 2 for QoR-15 score, 38 patients were in the no-preference-expression group and 165 in the preference-expression group. ^¶^Among patients followed at 1 month for EQ-5D-5L VAS, 31 patients were in the no-preference-expression group and 134 in the preference-expression group.

Outcomes	Preferenceexpression (*n*=177)	No preferenceexpression (*n*=43)	Mean/median/riskdifference (95% CI)(unadjusted effect)	*P*-value(unadjusted)
Secondary outcomes				
Bradycardia	1 (0.6)	1 (2.3)	−1.8 (−11.5 to 1.4)	0.353
Hypoxaemia				
PACU	88 (49.7)	26 (60.5)	−10.8 (−25.8 to 5.8)	0.206
POD 1	9 (5.1)	3 (7.1)	−2.0 (−14.1 to 4.3)	0.705
PONV				
PACU	13 (7.3)	2 (4.7)	2.7 (−8.5 to 8.6)	0.741
POD 1	12 (6.9)	3 (7.5)	−0.6 (−13.3 to 6.3)	1.000
Opioid consumption				
PACU	11.0 [10.0]	10.3 [13.1]	0.7 (−4.2 to 2.9)	0.714
POD 1	12.1 [19.7]	8.8 [16.7]	3.3 (−9.7 to 3.1)	0.314
Bauer patient satisfaction∗				
Information	110 (62.5)	23 (53.5)	9.0 (−6.8 to 25.1)	0.433
Emergence	72 (41.1)	14 (32.6)	8.6 (−7.9 to 22.7)	0.334
Pain therapy	64 (36.4)	9 (20.9)	15.4 (−0.4 to 27.4)	0.094
PONV	58 (33.0)	13 (30.2)	2.7 (−13.5 to 16.4)	0.693
Anaesthesia care	108 (61.4)	23 (53.5)	7.9 (−7.9 to 24.0)	0.604
Overall satisfaction^†^	8.8 [1.3]	8.1 [1.7]	−0.6 (−0.2 to 1.1)	0.009
QoR-15 score on POD 2^‡^	115.6 [16.2]	103.1 [23.4]	12.6 (6.3–18.9)	<0.001
EQ-5D-5L VAS^¶^	82.7 [13.5]	81.6 [12.7]	1.1 (−4.2 to 6.3)	0.683

Patient satisfaction scores were similar between groups on POD 1 across the Bauer questionnaire domains, except for slightly higher overall satisfaction in the preference group (8.8 [1.3] *vs* 8.1 [1.5]; *P*=0.009) (Table 4). Participants who expressed a preference were more likely to request the same anaesthetic strategy for future anaesthesia (86.4% *vs* 47.7%; *P*<0.001).

On POD 30, EQ-5D-5L health-related quality of life was similar between groups (82.7 [13.5] *vs* 81.6 [12.7]; *P*=0.680). Detailed outcomes for the four groups are presented in Supplementary Table 6.

### Exploratory and safety outcomes

There were no group differences in intraoperative hypotension, postoperative complications, readmissions, or DAH30. The incidence of moderate or severe pain and hospital length of stay were likewise similar (Supplementary Table 1).

## Discussion

In this prospective partially randomised patient-preference trial, participants who expressed a preference for intraoperative opioid management reported higher QoR-15 scores than those who did not express a preference and were randomly allocated. This association persisted after adjustment for anaesthetic strategy and prespecified clinical covariates. Importantly, this finding should be interpreted as an association with preference expression rather than as evidence of a causal effect of being allowed to choose or of shared decision making itself. All participants received the same decision aid and structured discussion, and preference expression was not randomised. Therefore, the observed association may reflect characteristics associated with the willingness or ability to express a treatment preference, including expectations, decisional certainty, self-efficacy, or greater engagement in care.

The adjusted between-group difference in POD 1 QoR-15 was below the 8-point MCID^40^ referenced in the original study protocol, although more recent work has proposed a threshold of approximately 6 points.^41^ Importantly, the CI encompassed effects ranging from very small to clearly clinically important, reflecting the limited precision associated with the relatively small no-preference-expression group. Therefore, the clinical importance of the observed association remains uncertain. Sensitivity analyses yielded similar point estimates but wider CIs and attenuated statistical evidence, further supporting cautious interpretation.

Our findings complement previous work examining the potential influence of patient choice on perioperative outcomes. Carvalho and colleagues^16^ reported that offering women undergoing Caesarean delivery a choice of postoperative analgesic regimen did not significantly reduce opioid consumption or pain scores, although patient satisfaction was greater among those offered a choice. Our study extends these observations by examining overall postoperative QoR rather than analgesic outcomes alone. Together, these findings suggest that patient choice and preference expression may be more closely associated with patient-reported experiences than with traditional analgesic or physiological outcomes. However, because preference expression was not randomised in the present study, these findings should not be interpreted as demonstrating that providing choice itself improves postoperative recovery.

Several mechanisms may explain the association between preference expression and postoperative recovery. Greater involvement in treatment decisions may influence expectations, perceived control, and satisfaction with care. Participants expressing a preference may also represent a population with greater self-efficacy or engagement with healthcare decisions.^1^ In addition, the opportunity to express a preference may have generated contextual effects that influenced recovery, given that evidence from randomised trials suggests that contextual factors can contribute substantially to overall treatment response, regardless of the specific effects of the intervention.^42^ This consideration is particularly relevant because QoR-15 is a patient-reported outcome. Participants expressing a preference were aware that they received their preferred strategy, whereas participants undergoing randomisation were intended to remain blinded to treatment allocation. Consequently, positive expectations, satisfaction with receiving one’s preferred treatment, or other contextual effects may have contributed to the observed difference in QoR-15. Such effects may themselves constitute meaningful components of the patient’s recovery experience, but they preclude attributing the observed difference to physiological recovery alone.

Interestingly, the observed differences were primarily driven by the physical independence domain of the QoR-15 score rather than by differences in complications or other objective clinical outcomes. This pattern may reflect a more favourable perception of early functional recovery among participants who expressed a preference. More broadly, it suggests that preference expression may influence how participants perceive and experience their recovery rather than substantially altering physiological recovery itself. Such an interpretation is consistent with contemporary models of patient-centred care, which emphasise the importance of patient autonomy, participation in decision making, and expectation management as determinants of healthcare experiences and outcomes.^1^^,^^43^

Interestingly, most participants who expressed a preference selected OBA rather than OFA. This finding contrasts with the increasing emphasis on opioid avoidance in contemporary perioperative medicine and suggests that patient perceptions regarding intraoperative opioids may differ from those of clinicians. The frequent opioid prescriptions and higher postoperative pain reported in the United States may further contribute to these perceptions.^44^^,^^45^ Overall, these observations suggest that patient preferences may be influenced as much by societal narratives and individual expectations as by objective assessments of risks and benefits. Importantly, these findings suggest that patient preferences regarding anaesthesia may not always align with prevailing assumptions among clinicians regarding opioid avoidance.

The association with preference expression should also be clearly distinguished from the pharmacological effects of the anaesthetic strategy itself. PERFECT was primarily designed to evaluate preference expression *vs* no preference expression and was not primarily powered to establish the comparative effectiveness of OFA *vs* OBA. Furthermore, anaesthetic strategy was randomised only among participants without a preference, whereas participants expressing a preference selected their treatment. Consequently, comparisons between OFA and OBA across the entire cohort are susceptible to treatment-selection confounding and should be considered exploratory. Therefore, the higher QoR-15 scores observed among participants expressing a preference should not be interpreted as evidence favouring either OFA or OBA.

Secondary outcomes were generally similar between groups. Although overall satisfaction scores were slightly higher among participants expressing a preference, differences in complications, hospital length of stay, readmissions, and health-related quality of life at 30 days were not observed. Importantly, the study was not powered to detect differences in these outcomes. The relative absence of differences in these more objective clinical outcomes further supports caution in interpreting the higher QoR-15 scores as evidence of improved physiological recovery.

Several limitations should be acknowledged. First, preference expression was not randomised, a recognised limitation of partially randomised patient-preference designs that introduces the possibility of residual confounding and selection bias.^46^ Participants who expressed a preference may have had higher levels of healthcare engagement, self-efficacy, patient activation, decisional certainty, or treatment expectations, characteristics that were not formally measured and could independently influence postoperative recovery. Therefore, preference expression may serve as a surrogate marker of broader patient engagement rather than as the direct mechanism responsible for the observed association. Consequently, the observed association may reflect differences in participant characteristics rather than an effect of preference expression itself. Indeed, individuals willing to express a treatment preference may represent a distinct behavioural phenotype characterised by greater engagement in healthcare decision making. Although several measured baseline characteristics were comparable between groups, the absence of identified baseline predictors of preference expression does not establish exchangeability between groups or rule out residual confounding by unmeasured characteristics. Second, the study was conducted in two academic centres among participants undergoing minimally invasive surgery, and most participants (89.1%) had previously undergone general anaesthesia. These characteristics may limit the generalisability of our findings, particularly to anaesthesia-naïve patients, other surgical populations, and different healthcare settings. Third, group sizes were substantially imbalanced because most participants expressed a preference, which reduced statistical precision and limited power for some comparisons. Fourth, the present study did not distinguish whether the expression of preference reflected decisional confidence, patient activation, expectations regarding treatment, health literacy, or other psychological constructs. Future studies should incorporate validated instruments to better characterise these potentially important mediators. Fifth, the primary outcome was patient reported and therefore susceptible to expectation and reporting effects, particularly because blinding varied by preference status, as discussed earlier. Consequently, the observed association may partly reflect positive treatment expectations, satisfaction with receiving one’s preferred strategy, or other contextual (placebo-like) effects rather than differences in the physiological effects of the anaesthetic strategy itself. This possibility should be considered when interpreting the present findings. Importantly, these expectation-related effects are not necessarily a source of measurement error but may represent clinically meaningful components of patient-centred recovery. Future studies should incorporate validated measures of treatment expectations and patient activation to better understand these mechanisms. Although follow-up was high for POD 2 and similar between preference groups, additional patient-reported outcomes were missing at later time points. Measured baseline characteristics did not differ significantly between participants with and without follow-up; nevertheless, differences in unmeasured characteristics and some degree of attrition bias cannot be excluded. Sixth, intraoperative protocol compliance was incomplete, reflecting the pragmatic nature of the study and contemporary multimodal analgesia practices. The per-protocol analysis yielded a larger effect estimate than the primary modified ITT analysis; however, it should be interpreted with caution because the exclusion of participants with protocol deviations may introduce additional selection bias. Accordingly, the modified ITT analysis was considered primary, and the per-protocol analysis was considered supportive only. Finally, some patient-reported outcomes were unavailable at later follow-up time points, and longer-term patient-centred outcomes were not evaluated.

### Implications for clinical practice

Contemporary perioperative care increasingly emphasises patient-reported outcomes and shared decision making,^3^ yet anaesthetic management remains largely clinician directed.^9^ Our findings suggest that expressing a treatment preference is associated with differences in early patient-reported recovery, independent of the specific anaesthetic strategy included in the adjusted model. However, they do not establish that actively providing treatment choice or shared decision making improves recovery. Preference expression may instead identify participants with different expectations, decisional certainty, self-efficacy, or engagement with their care. Distinguishing these possibilities will require a study in which the opportunity to choose is itself randomised. Therefore, future pragmatic trials should compare an approach in which participants are explicitly offered a choice with one in which treatment is assigned without choice, while measuring treatment expectations, patient activation, decisional conflict, and other potential psychological mediators. Such studies could determine whether facilitating patient participation itself improves postoperative recovery or whether preference expression primarily identifies individuals predisposed to report better recovery.

### Conclusion

In this prospective partially randomised patient-preference trial, expressing a preference for intraoperative opioid management was associated with higher early postoperative QoR-15 scores than no preference expression, followed by random allocation. The observed association should not be interpreted as demonstrating a causal effect of treatment preference, shared decision making, or either anaesthetic strategy. Residual confounding, differences in patient expectations and engagement, and the subjective nature of the primary outcome remain important alternative explanations. These findings support further investigation of whether actively facilitating patient preference can improve patient-reported postoperative recovery.

## Authors’ contributions

Conceptualisation: YG, AJ, PF, NMB

Methodology: YG, AJ, PF, TG, NMB

Formal analysis: TG

Investigation: all authors

Data curation: YG

Writing—original draft: YG, AJ

Writing—review and editing: all authors

Supervision: AJ, NMB

Project administration: YG, NMB

## Funding

National Institutes of Health (T32GM148369) to NMB.

## Declaration of interests

PF is supported by the European Society of Anaesthesiology and Intensive Care (ESAIC) for the Pain and Opioids After Surgery and the Euro-Periscope Research Groups (IDs ESAIC_GR_2021_PF, ESAIC_RG_PAND, and ESAIC_RG_EP) and received advisory board/speaker fees from Grünenthal, GE HealthCare, and Oncomfort. YG receives speaker fees from GE HealthCare and received personal financial support from the Phillip Foundation and Nîmes University Hospital for his UCLA research fellowship. Maxime Cannesson and Alexandre Joosten are consultants for Edwards Lifesciences, Irvine, CA, USA. The remaining authors declare that they have no conflicts of interest.
