# Supplementary material for: Patient preference for intraoperative opioid strategy and early postoperative recovery after noncardiac surgery: a prospective partially randomised patient-preference trial (the PERFECT trial)
Source: BJA Open. 2026 Oct 26;20:100867. doi: 10.1016/j.bjao.2026.100867 (PMC13734914; doi:10.1016/j.bjao.2026.100867)
Supplement: Supplementary file 1 [file mmc1.docx]

**Information for Participants: OBA and OFA**

**Understanding Your Anesthesia Options:**

- You have been invited to participate in a study evaluating whether your choice, or lack of choice, of anesthesia strategy may affect your early recovery after surgery
- We’ll discuss together the two possible general anesthesia techniques you may receive for your surgery:

**OBA (Opioid-Based Anesthesia): the traditional method**

**OFA (Opioid-Free Anesthesia): the alternative approach**

- This is the same information we give to everyone in our study, with clear and balanced information to help you make an potential decision about anesthesia strategy.
- We use the latest medical knowledge to give you clear and honest information, with no outside influence or hidden interests.

### **Shared Points for Both Methods: Whatever the Chosen Technique**

- You **won’t wake up** during the surgery
- You won’t hear, feel, or remember anything during the operation
- This **won’t affect** your surgical results; your surgeon is aware of both methods.
- Pain control after surgery is **similar** between both methods.

**Safety:**

- During general anesthesia, it is very common for your blood pressure and heart rate to fluctuate.
- Both options are safe, and we continuously monitor your vital signs (blood pressure, heart rate, breathing). Any changes are treated immediately.
- All medications used are FDA-approved and will be eliminated from your body within a few hours

**Option 1: Opioid-Based Anesthesia (OBA)**

- **What is it?**
  The usual way of giving anesthesia worldwide, using strong pain medicines called opioids, like Fentanyl.
- **Benefits:**
  - Well-known and widely used.
  - Many doctors and nurses are experienced with this
  - Proven effective for pain control during surgery.
- **Possible downsides:**
  - May cause **nausea**, **vomiting**, or **shivering** after surgery.
  - Rare chance of **constipation**, **urinary retention**, or **future dependence on opioids**.

**Option 2: Opioid-Free Anesthesia (OFA)**

- **What is it?**
  An alternative approach avoiding Fentanyl during your operation, using other medicines instead like Lidocaine, Ketamine, etc.
- **Benefits:**
  - Reduces significantly nausea and vomiting after surgery (compared to OBA)
  - No direct risk of opioid dependence.
- **Possible downsides :**
  - Requires careful monitoring by your doctors.
  - Less commonly used than OBA.

**What We Don’t Fully Know About OFA vs OBA**

- Differences in postop complications rate, long-term pain or overall recovery.
- Differences in patient satisfaction/experience over time.

**Your Options:**

- You can **choose OBA** (the traditional approach).
- You can **choose OFA** (the alternative approach).
- If you **don’t have a preference**, you can let us choose randomly (like a coin toss). In this case, you won’t know which option was used, just like in most of your previous surgeries.
- You will make your decision on the day of surgery, but feel free to ask questions now or later.

**Support for Your Decision :**

- Family members or close ones can be part of the discussion if you wish.
- We also value your feedback about this process—it helps us improve care for future patients.

**How to Make Your Decision?**

**Now that you know more about the two anesthesia options, take a moment to think about what matters most to you, including your concerns about side effects or past anesthesia experience.**

**Feel free to ask any questions to feel confident in your choice.**

### **Final Points:**

- No matter what you choose, we’ll give you the best care possible.
- You can **change your mind** anytime, even on the day of surgery.
- We’ll review your choice again tomorrow.
- Thank you for listening and helping us learn more about anesthesia methods.

## **Quick Quiz: Did You Understand the Differences?**

- **True or False:** Both OBA and OFA provide similar levels of pain control.
- **True or False:** OFA is better to eliminates post-surgical nausea and vomiting.
- **Multiple Choice:** Which anesthesia method is traditionally more widely used?
  a) OBA
  b) OFA
  c) Both are equally common

If you are unsure about any answer, feel free to ask your anesthesia team !

Responses: 1T; 2T; 3OBA
