# Supplementary material for: Patient preference for intraoperative opioid strategy and early postoperative recovery after noncardiac surgery: a prospective partially randomised patient-preference trial (the PERFECT trial)
Source: BJA Open. 2026 Oct 26;20:100867. doi: 10.1016/j.bjao.2026.100867 (PMC13734914; doi:10.1016/j.bjao.2026.100867)
Supplement: Supplementary file 2 [file mmc2.docx]

| **Supplementary Table 1. Baseline Characteristics** | | | | |
| --- | --- | --- | --- | --- |
|  | **Preference Expression** | | **No Preference Expression** | |
| **Characteristics** | **OFA-P**  **(N=49)** | **OBA-P**  **(N=128)** | **OFA-R**  **(N=21)** | **OBA-R**  **(N=22)** |
| Age | 56.4 ± 16.0 | 55.3 ± 14.5 | 53.5 ± 15.9 | 49.0 ± 13.9 |
| Female sex | 32 (65.3%) | 76 (59.4%) | 16 (76.2%) | 15 (68.2%) |
| Weight | 77.2 ± 18.7 | 81.9 ± 17.9 | 83.0 ± 28.0 | 89.9 ± 22.3 |
| Body mass index, kg/m²  College education or greater | 26.9 ± 6.1  39 (83%) | 28.7 ± 6.1  96 (76.8%) | 29.0 ± 8.0  19 (95%) | 31.7 ± 7.5  15 (71.5%) |
| *Ethnicity* |  |  |  |  |
| White  Hispanic  Asian  African American  Others | 36 (73.5%)  4 (8.2%)  6 (12.2%)  0  3 (6.1%) | 82 (65.1%)  15 (11.9%)  15 (11.9%)  6 (4.8%)  8 (6.3%) | 10 (47.6%)  4 (19.0%)  2 (9.5%)  1 (4.8%)  4 (19.0%) | 12 (54.5%)  5 (22.7%)  2 (9.1%)  1 (4.5%)  2 (9.1%) |
| *Type of surgery* |  |  |  |  |
| Gastrointestinal  Colorectal  Hernia repair  Urologic  Gynaecologic | 4 (8.2%)  2 (4.1%)  7 (14.3%)  13 (26.5%)  23 (46.9%) | 17 (13.3%)  8 (6.2%)  20 (15.6%)  42 (32.8%)  42 (32.8%) | 6 (28.6%)  4 (19.0%)  3 (14.3%)  3 (14.3%)  6 (28.6%) | 6 (27.3%)  1 (4.5%)  3 (13.6%)  3 (13.6%)  10 (45.5%) |
| *ASA score* |  |  |  |  |
| I  II  III  IV | 5 (10.2%)  24 (49.0%)  20 (40.8%)  0 | 7 (5.5%)  65 (50.8%)  55 (43.0%)  1 (0.8%) | 2 (9.5%)  7 (33.3%)  12 (57.1%)  0 | 1 (4.5%)  13 (59.1%)  8 (36.4%)  0 |
| *Medical history* |  |  |  |  |
| High blood pressure  Dyslipidaemia  Coronary artery disease  Diabetes mellitus  COPD / Asthma  Sleep apnoea  Chronic renal failure  Active cancer  Substance use disorder  Previous chronic pain  Previous chronic opioid use  Previous general anaesthesia  Previous abdominal surgery  Previous PONV  *Current medications*  Antidepressant  Anxiolytic  Non opioid analgesics  *Preoperative anxiety related to:*  General anaesthesia *  Opioid exposure | 17 (34.7%)  17 (34.7%)  7 (14.3%)  4 (8.2%)  4 (8.2%)  5 (10.2%)  3 (6.1%)  19 (38.8%)  15 (30.6%)  7 (14.3%)  7 (14.3%)  42 (85.7%)  32 (66.7%)  10 (20.4%)  7 (14.3%)  3 (6.1%)  21 (42.9%)  25 (51%)  32 (65.3%) | 57 (44.5%)  66 (51.6%)  16 (12.5%)  21 (16.4%)  17 (13.3%)  20 (15.6%)  4 (3.1%)  54 (42.2%)  35 (27.6%)  9 (7.1%)  16 (12.6%)  115 (89.8%)  74 (57.8%)  19 (14.8%)  21 (16.4%)  11 (8.6%)  46 (35.9%)  80 (62.5%)  74 (57.8%) | 7 (33.3%)  7 (33.3%)  2 (9.5%)  4 (19.0%)  3 (14.3%)  4 (19.0%)  1 (4.8%)  2 (9.5%)  6 (28.6%)  3 (14.3%)  2 (9.5%)  20 (95.2%)  13 (61.9%)  4 (19.0%)  5 (23.8%)  3 (14.3%)  4 (19.0%)  13 (62%)  14 (66.6%) | 9 (40.9%)  10 (45.5%)  1 (4.5%)  5 (22.7%)  2 (9.1%)  3 (13.6%)  0  5 (22.7%)  4 (18.2%)  2 (9.1%)  2 (9.1%)  18 (81.8%)  10 (45.5%)  1 (4.5%)  4 (18.2%)  3 (13.6%)  7 (31.8%)  10 ( 45%)  11 (50%) |

Data are presented as mean (SD) or n (%).

Legend: ASA, American Society of Anaesthesiologists; COPD, Chronic Obstructive Pulmonary Disease; OBA, Opioid-Based Anaesthesia; OFA, Opioid-Free Anaesthesia; OBA-P, Opioid-Based Anaesthesia –Preference; OFA-P, Opioid-Free Anaesthesia – Preference; OBA-R, Opioid-Based Anaesthesia – Randomized; OFA-R, Opioid-Free Anaesthesia – Randomized; PONV, Postoperative Nausea and Vomiting; P, Preference; R, Randomized.

*Defined as any level of worry about receiving general anaesthesia (any response other than “not at all worried”).
