# Supplementary material for: Patient preference for intraoperative opioid strategy and early postoperative recovery after noncardiac surgery: a prospective partially randomised patient-preference trial (the PERFECT trial)
Source: BJA Open. 2026 Oct 26;20:100867. doi: 10.1016/j.bjao.2026.100867 (PMC13734914; doi:10.1016/j.bjao.2026.100867)
Supplement: Supplementary file 3 [file mmc3.docx]

| **Predictor** | **Univariable**  **OR (95% CI)** | **P value** | **Multivariable OR (95% CI)** | **P value** |
| --- | --- | --- | --- | --- |
| *Preoperative* |  |  |  |  |
| Age (per year)  Sex (male vs female)  Substance Abuse History  Chronic Pain History  History of Opioid Use  PONV History  Previous Abdominal Surgery  *Surgery type*  Gastro-intestinal  Colorectal  Urologic  Gynaecologic  Hernia repair | 1.02 (1.00 to 1.04)  0.61 (0.28 to 1.23)  1.31 (0.62 to 2.98)  0.76 (0.28 to 2.44)  1.47 (0.53 to 5.21)  1.49 (0.58 to 4.60)  1.32 (0.67 to 2.58)  0.35 (0.16 to 0.80)  0.46 (0.15 to 1.53)  2.78 (1.18 to 7.67)  0.98 (0.50 to 1.98)  1.11 (0.45 to 3.15) | 0.086  0.180  0.498  0.613  0.503  0.441  0.421  0.010  0.172  0.029  0.953  0.830 | 1.02 (0.99 to 1.04)  0.69 (0.25 to 1.98)  1.13 (0.49 to 2.76)  0.66 (0.20 to 2.40)  1.18 (0.36 to 4.75)  1.81 (0.61 to 6.48)  1.28 (0.59 to 2.77)  0.14 (0.01 to 1.80)  0.09 (0.00 to 1.37)  0.36 (0.01 to 6.03)  0.26 (0.01 to 4.23)  0.26 (0.01 to 3.68) | 0.220  0.490  0.787  0.503  0.799  0.318  0.533  0.138  0.099  0.489  0.361  0.324 |

**Supplementary Table 2: Univariable and multivariable logistic regression analyses of factors associated with anaesthesia preferences.**

Values are presented as odds ratios (OR) with 95% confidence intervals (CI).

Legend: PONV: Post-operative Nausea and Vomiting.
