# Supplementary material for: Patient preference for intraoperative opioid strategy and early postoperative recovery after noncardiac surgery: a prospective partially randomised patient-preference trial (the PERFECT trial)
Source: BJA Open. 2026 Oct 26;20:100867. doi: 10.1016/j.bjao.2026.100867 (PMC13734914; doi:10.1016/j.bjao.2026.100867)
Supplement: Supplementary file 4 [file mmc4.docx]

| **Supplementary Table 3: sensitivity analysis for the primary outcome** | | | | | | | |
| --- | --- | --- | --- | --- | --- | --- | --- |
| **Spe** | **Weights** | **SE type** | **Contrast** | **Estimate** | **95% CI** | **p-value** | **Comment** |
| **Primary** | Equal | Model-based | Preference vs  No Preference | 7.1 | (0.27 – 13.93) | 0.0416 | *Prespecified main analysis, statistically significant* |
| **Primary** | Equal | Robust HC3 | Preference vs  No Preference | 7.1 | (−0.87 – 15.08) | 0.081 | *Slightly wider CI using heteroskedasticity-robust SEs* |
| **Primary** | Proportional | Robust HC3 | Preference vs  No Preference | 6.2 | (−1.77 – 14.18) | 0.127 | *Small attenuation when weighting by group size* |
| **Sensitivity**  **(+Block + Comorbidity)** | Proportional | Robust HC3 | Preference vs  No Preference | 6.1 | (−1.9 – 14.2) | 0.134 | *Same direction and magnitude after additional adjustment* |
| **Minimal parsimonious** | Equal | Model-based | Preference vs No Preference | 7.73 | (0.96 to 14.51) | 0.025 |  |
| **Minimal parsimonious** | Equal | Robust HC3 | Preference vs No Preference | 7.73 | (-0.19 to 15.66) | 0.056 |  |
| **Parsimonious + surgical category** | Equal | Model-based | Preference vs No Preference | 6.46 | (-0.48 to 13.40) | 0.068 |  |
| **Parsimonious + surgical category** | Equal | Robust HC3 | Preference vs No Preference | 6.46 | (-1.67 to 14.59) | 0.119 |  |
| **Excluding two Figure 2 outliers** | Equal | Model-based | Preference vs No Preference | 7.25 | (0.67 to 13.83) | 0.031 |  |
| **Excluding two Figure 2 outliers** | Equal | Robust HC3 | Preference vs No Preference | 7.25 | (−0.69 to 15.19) | 0.073 |  |

Values are presented as estimated mean differences with 95% confidence intervals.

“Sensitivity (+Block + Comorbidity)” refers to an additional model adjusted for the presence of a fascial plane block and for a composite comorbidity count, defined as the sum of six major preoperative conditions.

The minimal parsimonious model included preference expression, anaesthetic strategy, their interaction, age, sex, active cancer, and substance use disorder. The parsimonious + surgical category model additionally included the surgical-category indicator terms. All models were fitted to the same complete-case sample (N=213). The outlier sensitivity excluded the two boxplot-defined POD1 QoR-15 observations in the preference-expression group; both observations were retained in the prespecified primary analysis.
