# Supplementary material for: Patient preference for intraoperative opioid strategy and early postoperative recovery after noncardiac surgery: a prospective partially randomised patient-preference trial (the PERFECT trial)
Source: BJA Open. 2026 Oct 26;20:100867. doi: 10.1016/j.bjao.2026.100867 (PMC13734914; doi:10.1016/j.bjao.2026.100867)
Supplement: Supplementary file 5 [file mmc5.docx]

**Supplementary table 4:** **Multivariable linear regression models for the primary outcome**

| **Predictor** | **Primary model (equal weights, model-based SE)** | **p-value** | **Primary model (equal weights, robust HC3 SE)** | **p-value** | **Primary model (proportional weights, robust HC3 SE)** | **p-value** | **Sensitivity model (+ regional anesthesia + comorbidity count; proportional weights, robust HC3 SE)** | **p-value** |
| --- | --- | --- | --- | --- | --- | --- | --- | --- |
| **Preference (Preference vs No Preference)** | 4.67 (−4.13, 13.47) | 0.299 | 4.67 (−5.37, 14.71) | 0.362 | 4.72 (−4.10, 13.53) | 0.296 | 4.72 (−5.32, 14.75) | 0.357 |
| **Opioid-free anaesthesia (OFA vs OBA)** | −3.70 (−15.26, 7.86) | 0.531 | −3.70 (−18.06, 10.66) | 0.613 | −2.87 (−14.48, 8.74) | 0.628 | −2.87 (−17.50, 11.75) | 0.7 |
| **Preference × OFA interaction** | 4.86 (−8.37, 18.09) | 0.472 | 4.86 (−10.71, 20.43) | 0.541 | 4.53 (−8.70, 17.77) | 0.503 | 4.53 (−11.24, 20.30) | 0.573 |
| Age (years) | 0.21 (0.01, 0.41) | 0.039 | 0.21 (0.00, 0.42) | 0.046 | 0.14 (−0.08, 0.36) | 0.201 | 0.14 (−0.09, 0.38) | 0.23 |
| Sex | −1.40 (−8.96, 6.16) | 0.716 | −1.40 (−9.22, 6.42) | 0.725 | −0.25 (−7.95, 7.45) | 0.949 | −0.25 (−8.08, 7.58) | 0.95 |
| Substance abuse history | 8.20 (2.14, 14.26) | 0.009 | 8.20 (1.78, 14.63) | 0.012 | 8.20 (2.12, 14.29) | 0.009 | 8.20 (1.84, 14.57) | 0.012 |
| Chronic pain history | −7.45 (−16.89, 2.00) | 0.124 | −7.45 (−18.00, 3.11) | 0.167 | −7.36 (−16.80, 2.07) | 0.128 | −7.36 (−18.22, 3.50) | 0.184 |
| Prior opioid use | −4.27 (−12.73, 4.20) | 0.325 | −4.27 (−14.40, 5.87) | 0.409 | −4.69 (−13.20, 3.81) | 0.281 | −4.69 (−14.89, 5.50) | 0.367 |
| PONV history | −1.25 (−8.70, 6.21) | 0.744 | −1.25 (−9.32, 6.83) | 0.763 | −1.16 (−8.61, 6.28) | 0.76 | −1.16 (−9.38, 7.05) | 0.781 |
| Prior abdominal surgery | −3.01 (−8.55, 2.52) | 0.288 | −3.01 (−8.83, 2.81) | 0.31 | −3.55 (−9.13, 2.03) | 0.214 | −3.55 (−9.44, 2.35) | 0.239 |
| Length of surgery (min) | −0.03 (−0.07, 0.01) | 0.094 | −0.03 (−0.07, 0.00) | 0.086 | −0.03 (−0.07, 0.01) | 0.134 | −0.03 (−0.07, 0.01) | 0.125 |
| Gastro-Intestinal surgery | −17.98 (−40.43, 4.48) | 0.118 | −17.98 (−45.21, 9.25) | 0.196 | −17.90 (−40.46, 4.67) | 0.122 | −17.90 (−49.44, 13.65) | 0.266 |
| Colorectal surgery | −24.41 (−48.76, −0.07) | 0.051 | −24.41 (−55.73, 6.90) | 0.127 | −25.96 (−50.42, −1.50) | 0.039 | −25.96 (−61.80, 9.88) | 0.156 |
| Urologic surgery | −20.22 (−44.11, 3.67) | 0.099 | −20.22 (−49.38, 8.93) | 0.174 | −21.63 (−45.89, 2.64) | 0.082 | −21.63 (−54.89, 11.63) | 0.203 |
| Gynaecologic surgery | −14.84 (−38.93, 9.25) | 0.229 | −14.84 (−44.06, 14.38) | 0.32 | −15.33 (−39.69, 9.02) | 0.219 | −15.33 (−48.38, 17.72) | 0.363 |
| Hernia repair | −23.72 (−46.85, −0.59) | 0.046 | −23.72 (−51.39, 3.94) | 0.093 | −23.27 (−46.70, 0.15) | 0.053 | −23.27 (−55.01, 8.47) | 0.151 |
| Regional anaesthesia block | — | — | — | — | −0.88 (−9.03, 7.28) | 0.833 | −0.88 (−8.77, 7.01) | 0.828 |
| Comorbidity count | — | — | — | — | 2.23 (−0.65, 5.11) | 0.131 | 2.23 (−0.86, 5.33) | 0.158 |
| Preference vs no preference contrast | **7.1 (0.27 to 13.93)** | 0.042 | **7.1 (−0.87 to 15.08)** | 0.081 | **6.2 (−1.77 to 14.18)** | 0.127 | **6.1 (−1.90 to 14.18)** | 0.134 |

Legend: OFA: opioid free anaesthesia

Because the model includes a preference-expression × anaesthetic-strategy interaction, the main-effect coefficients are conditional effects within the corresponding reference stratum. The coefficient for Preference represents Preference versus No Preference within OBA, and the coefficient for OFA represents OFA versus OBA within the No-Preference group. The overall marginal contrasts averaged across strata are reported separately.
