# Supplementary material for: Patient preference for intraoperative opioid strategy and early postoperative recovery after noncardiac surgery: a prospective partially randomised patient-preference trial (the PERFECT trial)
Source: BJA Open. 2026 Oct 26;20:100867. doi: 10.1016/j.bjao.2026.100867 (PMC13734914; doi:10.1016/j.bjao.2026.100867)
Supplement: Supplementary file 6 [file mmc6.docx]

| **Supplementary Table 5. Outcomes according to the allocated groups** | | | | | |
| --- | --- | --- | --- | --- | --- |
|  | **Preference Expression** | | **No Preference Expression** | |  |
| **Outcomes** | **OFA-P**  **(N=49)** | **OBA-P (N=128)** | **OFA-R**  **(N=21)** | **OBA-R**  **(N=22)** | **P value**  **(unadjusted)** |
| *Primary outcome: QoR15 at POD1* |  |  |  |  |  |
| Total score  Subdimensions  Physical comfort  Pain management  Physical independence  Psychological support  Emotional state | 110.1 ± 18.4  36.3 ± 9.5  12.7 ± 3.6  12.1 ± 4.1  18.5 ± 2.7  30.6 ± 5.7 | 108.2 ± 18.2  34.9 ± 7.9  13.5 ± 4.0  11.3 ± 4.8  18.7 ± 1.9  30.1 ± 6.7 | 98.8 ± 25.0  31.8 ± 10.7  11.1 ± 4.8  10.4 ± 5.1  17.9 ± 3.0  27.5 ± 8.9 | 102.7 ± 22.4  33.6 ± 11.6  12.6 ± 3.6  9.3 ± 4.7  18.2 ± 2.2  29.0 ± 7.0 | 0.091  0.26  0.073  0.115  0.363  0.321 |
| *Secondary outcomes* |  |  |  |  |  |
| Bradycardia  Hypoxemia  PACU  POD1  PONV  PACU  POD1  Opioid consumption  PACU  POD1  Bauer patient satisfaction†  Information  Emergence  Pain therapy  PONV  Anaesthesia care  Overall satisfaction‡  QoR-15 score at POD2*  EQ-5D-5L VAS** | 0  19 (38.8%)  2 (4.1%)  4 (8.2%)  4 (8.2%)  9.1 ± 10.7  11.8 ± 27.9  37 (75.5%)  19 (39.6%)  21 (42.9%)  20 (40.8%)  33 (67.3%)  8.8 ± 1.4  115.1 ± 16.7  84.2 ± 10.9 | 1 (0.8%)  69 (53.9%)  7 (5.5%)  9 (7.0%)  8 (6.4%)  11.7 ± 9.6  12.2 ± 15.6  73 (57.5%)  53 (41.7%)  43 (33.9%)  38 (29.9%)  75 (59.1%)  8.8 ± 1.3  115.8 ± 16.1  82.1 ± 14.5 | 1 (4.8%)  10 (47.6%)  1 (4.8%)  1 (4.8%)  2 (9.5%)  6.2 ± 8.1  7.9 ± 12.4  13 (61.9%)  7 (33.3%)  3 (14.3%)  5 (23.8%)  12 (57.1%)  8.0 ± 1.7  100.4 ± 22.2  81.3 ± 13.0 | 0  16 (72.7%)  2 (9.5%)  1 (4.5%)  1 (5.3%)  14.3 ± 15.7  9.6 ± 20.3  10 (45.5%)  7 (31.8%)  6 (27.3%)  8 (36.4%)  11 (50.0%)  8.3 ± 1.6  105.8 ± 24.8  82.1 ± 12.7 | 0.286  0.056  0.834  0.97  0.885  0.038  0.774  0.13  0.424  0.275  0.344  0.758  0.062  0.001  0.819 |
| *Exploratory outcomes* |  |  |  |  |  |
| Hypotension episodes  Intraoperative vasopressors  Highest pain scores, NRS  PACU  POD1  POD2  Hospital LOS  Follow up at POD30  Complications  Readmissions  DAH30^$^ | 40 (83.3%)  35 (71.4%)  4.3 ± 2.7  4.6 ± 2.1  3.8 ± 1.9  1.0 [0.0 to 1.0]  0  0  49 (100.0%) | 90 (70.3%)  79 (61.7%)  5.1 ± 2.3  4.6 ± 2.2  3.8 ± 1.7  1.0 [0.0 to 1.0]  8 (6.3%)  8 (6.2%)  120 (93.8%) | 15 (71.4%)  13 (61.9%)  4.3 ± 2.6  5.2 ± 2.3  4.8 ± 2.1  1.0 [0.0 to 1.0]  1(4.8%)  1 (4.8%)  20 (95.2%) | 16 (72.7%)  14 (63.6%)  4.4 ± 2.0  4.2 ± 2.3  4.0 ± 1.5  0.5 [0.0 to 1.0]  2 (9.1%)  2 (9.1%)  20 (90.9%) | 0.376  0.681  0.172  0.481  0.169  0.874  0.707  0.162  0.415 |

The values are means ± SD, n (%) or medians [Q1 to Q3]

†Bauer questionnaire: only ‘very satisfied’ responses are presented. ‡Overall satisfaction is reported as a 0–10 numeric rating scale.

*Among patients followed at POD2 for QoR-15 score: 42 patients in OFA-P group, 123 in OBA-P group, 19 in OFA-R group and 19 in OBA-R group.

**Among patients followed at 1 month for EQ 5D 5L VAS: 40 patients in OFA-P group, 94 in OBA-P group, 17 in OFA-R group and 14 in OBA-R group.

^$^DAH30 is presented as the proportion of patients with 30 days

Legend: DAH 30: Days at home at 30days; LOS: Length of stay; NRS: Numeric rating scale; OBA-P, Opioid-Based Anaesthesia – Preference; OFA-P, Opioid-Free Anaesthesia – Preference; OBA-R, Opioid-Based Anaesthesia – Randomized; OFA-R, Opioid-Free Anaesthesia – Randomized; PACU: Post operative care unit; POD: Post operative day; PONV, Post Operative Nausea and Vomiting; QoR: Quality of Recovery; VAS: Visual analogue scale.
