# Supplementary material for: Patient preference for intraoperative opioid strategy and early postoperative recovery after noncardiac surgery: a prospective partially randomised patient-preference trial (the PERFECT trial)
Source: BJA Open. 2026 Oct 26;20:100867. doi: 10.1016/j.bjao.2026.100867 (PMC13734914; doi:10.1016/j.bjao.2026.100867)
Supplement: Supplementary file 7 [file mmc7.docx]

| **Supplementary Table 6. Exploratory outcomes** | | | | |
| --- | --- | --- | --- | --- |
| **Outcomes** | **Preference Expression**  **(N=177)** | **No Preference Expression**  **(N=43)** | **Mean/Median/Risk**  **difference (95% CI)**  **Unadjusted effect** | **P value (unadjusted)** |
| *Exploratory outcomes* |  |  |  |  |
| Intraoperative hypotension episodes  Intraoperative vasopressors  Intraoperative hypertension episodes  Intraoperative tachycardia episodes  SBP < 90mmHg in PACU  Highest pain scores, NRS  PACU  POD#1  POD#2  Hospital LOS  Follow up at POD#30  Complications  Readmissions  DAH30^$^ | 130 (73.9%)  114 (64.4%)  48 (27.1%)  3 (1.7%)  19 (10.7%)  4.8 ±2.5  4.6 ±2.1  3.8 ±1.8  0.8 ±0.9  8 (4.5%)  8 (4.5%)  169 (95.5%) | 31 (72.1%)  27 (62.8%)  13 (30.2%)  2 (4.7%)  8 (18.6%)  4.4 ±2.3  4.7 ±2.3  4.4 ±1.8  0.8 ±0.9  3 (7.0%)  3 (7.0%)  40 (93.0%) | 1.8% (-11.4% to 17.7%)  1.6% (-13.1% to 18.0%)  -3.1% (-19.2% to 10.5%)  -3.0% (-13.8% to 1.7%)  -7.9% (-22.4% to 2.5%)  0.5 (-0.3 to 1.3)  -0.1 (-0.9 to 0.6)  -0.6 (-1.2 to 0.1)  0.0 (-0.3 to 0.3)  -2.5% (-14.3% to 3.7%)  -2.5% (-14.3% to 3.7%)  2.5% (-3.7% to 14.3%) | 0.814  0.843  0.682  0.252  0.158  0.249  0.703  0.069  0.889  0.453  0.453  0.453 |

The values are means ± SD, n (%) or (95% CI)

^$^DAH30 is presented as the proportion of patients with 30 days

Legend: DAH 30: Days at home at 30 days; LOS: Length of stay; NRS: Numeric rating scale; PACU: Post-operative care unit; POD: Post-operative day; SBP, Systolic Blood Pressure
